# Intrinsically Unstructured Domain 3 of Hepatitis C Virus NS5A Forms a “Fuzzy Complex” with VAPB-MSP Domain Which Carries ALS-Causing Mutations

**DOI:** 10.1371/journal.pone.0039261

**Published:** 2012-06-13

**Authors:** Garvita Gupta, Haina Qin, Jianxing Song

**Affiliations:** 1 Department of Biological Sciences, Faculty of Science, National University of Singapore, Singapore, Republic of Singapore; 2 Department of Biochemistry, Yong Loo Lin School of Medicine, National University of Singapore, Singapore, Republic of Singapore; Spanish National Cancer Center, Spain

## Abstract

Hepatitis C virus (HCV) affects nearly 200 million people worldwide and is a leading factor for serious chronic liver diseases. For replicating HCV genome, the membrane-associated replication machinery needs to be formed by both HCV non-structural proteins including NS5A and human host factors. Recently NS5A has been identified to bind ER-anchored human VAP proteins and consequently this interaction may serve as a novel target for design of anti-HCV drugs. So far no biophysical characterization of this interaction has been reported. Here, we dissected the 243-residue VAPB into 4 and 447-residue NS5A into 10 fragments, followed by CD and NMR characterization of their structural properties. Subsequently, binding interactions between these fragments have been extensively assessed by NMR HSQC titration which is very powerful in detecting even very weak binding. The studies lead to three important findings: 1). a “fuzzy complex” is formed between the intrinsically-unstructured third domain (D3) of NS5A and the well-structured MSP domain of VAPB, with an average dissociation constant (Kd) of ∼5 µM. 2). The binding-important residues on both NS5A-D3 and VAPB-MSP have been successfully mapped out, which provided experimental constraints for constructing the complex structure. In the complex, unstructured D3 binds to three surface pockets on one side of the MSP structure. Interestingly, two ALS-causing mutations T46I and P56S are also located on the D3-MSP interface. Moreover, NS5A-D3, FFAT-containing proteins and EphA4 appear to have overlapped binding interfaces on the MSP domain. 3). NS5A-D3 has been experimentally confirmed to competes with EphA4 in binding to the MSP domain, and T46I mutation of MSP dramatically abolishes its binding ability to D3. Our study not only provides essential foundation for further deciphering structure and function of the HCV replication machinery, but may also shed light on rationalizing a recent observation that a chronic HCV patient surprisingly developed ALS-like syndrome.

## Introduction

Hepatitis C virus (HCV), first discovered in 1989, is a member of the *Flaviviridae* family of enveloped, positive-strand RNA viruses [1], [2]. It is the major causative agent of non-A, non-B hepatitis and about 200 million people are infected with HCV worldwide [3]. HCV is a main risk factor for the development of serious chronic liver diseases including cirrhosis and hepatocellular carcinoma [4]. HCV has a genome approximately 9.6 kb in length, which encodes a single long polyprotein of about 3,000 amino acids, which is subsequently processed into 10 individual proteins by viral and cellular proteases [5]–[9]. Replication of positive-strand RNA viruses appears to always involve certain intracellular membrane structures, such as the endoplasmic reticulum (ER), Golgi apparatus, endosome, and lysosome [10]. Replication of HCV initiates immediately after translation and processing of the viral protein and all of HCV gene products remain associated with intracellular membranes [11]–[17]. The membrane-associated replication machinery copies the genome RNA into a negative-strand intermediate, which is then used to generate additional positive-strand RNAs for subsequent rounds of translation and packaging into virus particles. HCV nonstructural proteins including NS3, NS4A, NS4B, NS5A, and NS5B appear to be the key components of the RNA replication machinery but the exact details are poorly understood, such as the identities of the host factors and detailed interactions among them.

So far there is no clinically proven vaccine and the most common therapy is based on with a combination of pegylated interferon-alpha (PEG-IFNa) and ribavirin (RBV), which only has a success rate of ∼50% as well as severe side effects [18], [19]. As a consequence, identification of novel targets for design of HCV antiviral drugs is urgently demanded [20]–[23]. At present, the most popular targets are two enzymes: the NS3/4A serine protease and the NS5B RNA-dependent RNA polymerase as they are amenable to the development of biochemical assays for inhibitor screening [24]. However, they appear also to have a considerable disadvantage: low genetic barriers to drug resistance [24], [25]. Therefore, it may hold promising potential to develop drugs to target non-enzymatic components required for RNA replication. Indeed, although the underlying mechanism by which cyclophilins contribute to viral replication remains unknown, cyclophilin A, a molecular chaperone catalysing the cis-trans isomerization of proline residues, has been demonstrated to be an important drug target for therapy of chronic hepatitis C [26], [27].

Recently, inhibitors with a potent clinical effect have also been identified to target the HCV nonstructural 5 (NS5A) protein, which has no enzymatic activity [28]–[31]. NS5A consisting of 447 residues is a critical component of HCV replication [32] and is additionally involved in modulation of cell signaling pathways, interferon response, pathogenesis and apoptosis regulation [33]. As shown in Figure S1, it is composed of three domains connected by flexible linkers, domain 1 (33–202), domain 2 (251–342) and domain 3 (359–447). The crystal structures of domain 1 has been determined [34], [35] while both domain 2 [36], [37] and domain 3 [38], [39] have been characterized to be largely disordered by NMR spectroscopy.

**Figure 1:**
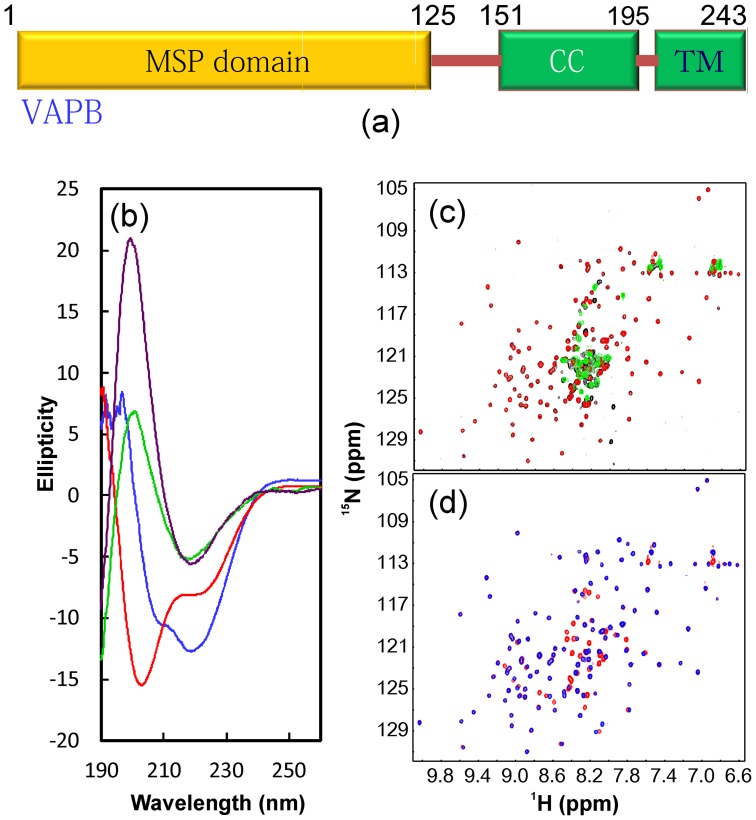
CD and NMR characterization of VAPB domains. (a). Domain organization of the 243-residue human VAPB protein consisting of the major sperm protein (MSP), coiled coil (CC) and transmembrane (TM) domains. (b). Far UV CD spectra of VAPB(1–195) (blue); VAPB(1–150) (green); VAPB(1–125) (brown) and VAPB-CC(151–195) (red). (c). Superimposition of ^1^H-^15^N NMR HSQC spectra of VAPB(1–195) (black); VAPB(1–150) (red) and VAPB-CC(151–195) (green). (d). Superimposition of ^1^H-^15^N NMR HSQC spectra of VAPB(1–150) (red); VAPB(1–125) (blue).

Remarkably, NS5A has been recently identified to bind human VAP family proteins VAPA/VAPB and this interaction appears to anchor the RNA replication machinery onto the ER for the replication of the HCV genome [15]–[17]. In particular, overexpression of VAPB significantly enhanced the expression of NS5A and NS5B, as well as the replication of HCV RNA [15], [17]. On the other hand, NS5A hyperphosphorylation disrupts its interaction to VAPA thus negatively regulating viral RNA replication [16]. The human VAP family proteins were initially identified as homologues of vesicle-associated membrane protein (VAMP)-associated protein (VAP) with a size of 33 kDa in *Aplysia californica*, including VAPA, VAPB, VAPC and several newly-identified spliced variants [40]–[42]. VAPA and VAPB are ∼60% identical in sequence and composed of three conserved domains, namely, an N-terminal immunoglobulin-like β sheet domain that is 22%identical in sequence to the major spermprotein (MSP), a central coiled-coil domain, and a C-terminal transmembrane domain (Figure 1a). VAP proteins are ubiquitously expressed, type II integral membrane proteins that localize to the endoplasmic reticulum (ER) and pre-Golgi intermediates [43]. Moreover, VAP proteins have been shown to target lipid-binding proteins carrying a short motif containing two phenylalanines in an acidic tract (FFAT motif) to the ER [44]–[46]. The FFAT-motif consists of the consensus amino acid sequence EFFDAxE, which was conserved in several lipid-binding protein families implicated in the transfer of lipids between the ER and other organelles, such as the Golgi, endosomes, and plasma membrane [47], [48]. The VAP proteins also interact with intracellular proteins, including Nir1, Nir2, and Nir3 via the FFAT motif which differentially affects the organization of the ER [49]. Most recently, it was also shown that the VAPB-MSP domain also serves as a ligand for Eph receptors [50], [51]. Strikingly, two point mutations P56S and T46I in the VAPB MSP domain have been identified to lead to familial amyotrophic lateral sclerosis with rapid progression or late onset spinal muscular atrophy [52], [53].

So far no detailed structural and binding characterization has been reported for the NS5A-VAPB interactions. On the other hand, such knowledge is essential for developing novel strategies to treat HCV infection [54]. In the present study, we first dissected both NS5A and VAPB into a large set of domains/fragments, followed by extensive structural and binding characterizations with both CD and NMR spectroscopy. The binding residues have been successfully mapped out by NMR HSQC titrations on both NS5A and VAPB, thus allowing the construct of the complex structure. Notably, NS5A binds to the VAPB surfaces carrying ALS-causing P56S and T46I mutants, which have been previously characterized to be also critical for binding FFAT-containing proteins and Eph receptors [44], [46], [51].

## Results

### Cloning, expression and characterization of dissected domains/fragments of VAPB

To map out the binding regions for the NS5A-VAPB interaction, in the present study we first dissected the 243-residue human VAPB into 4 domains/fragments (Figure 1a and Table S1): VAPB(1–195) only with the transmembrane fragment deleted, VAPB(1–150), VAPB(1–125) and the coiled-coil domain designated as VAPB-CC(151–195). Subsequently we succeeded in subcloning them in His-tagged expression vector, which were over-expressed in *E. coli* BL21 cells. The recombinant proteins were purified by Ni^2+^-affinity columns and then cleaved by thrombin, which were further purified by FPLC on a Superdex-200 gel-filtration column or HPLC on RP (reverse-phase) columns (Table S1).

As seen in Figure 1b, VAPB(1–195) has a far-UV CD spectrum for a protein containing both α-helix and β-sheet secondary structures. On the other hand, VAPB(1–150) has a far-UV CD spectrum similar to that of VAPB(1–125) which was previously characterized to adopt a β-dominant MSP fold [46]. This observation implies that the extra C-terminal 25 residues in VAPB(1–150) is largely unstructured and its presence does not alter the MSP-fold. Indeed, in a NMR structure (PDB ID: 2CRI) of the mouse VAPA(1–140) fragment, residues 125–140 are predominantly disordered. On the other hand, VAPB-CC has a far-UV CD spectrum characteristic of a helical conformation (Figure 1b) which is commonly observed on other coiled coil domains.

We then ^15^N-isotople labeled these fragments and acquired their ^1^H-^15^N NMR HSQC spectra. As shown in Figure 1c, VAPB(1–195) has a well-dispersed HSQC spectrum, indicating that it contains a well-folded domain. Interestingly, the HSQC spectrum of VAPB(1–195) appears to be almost the superimposition of the spectra of isolated VAPB(1–150) and VAPB-CC (Figure 1c), suggesting that VAPB(1–150) and VAPB-CC have no significant packing interaction in VAPB(1–195). Furthermore, as shown in Figure 1d, most HSQC peaks of VAPB(1–125) are also superimposable to those of VAPB(1–150), indicating that the C-terminal 25 residues of VAPB(1–150) have no significant packing with the MSP fold assumed by the N-terminal 125 residues [46]. Taken together, CD and NMR results demonstrate that VAPB(1–195) is composed two structural domains, namely the well-folded β-dominant MSP fold and helical VAPB-CC linked by the flexible loop.

### Cloning, expression and characterization of dissected domains/fragments of NS5A

**Figure 2:**
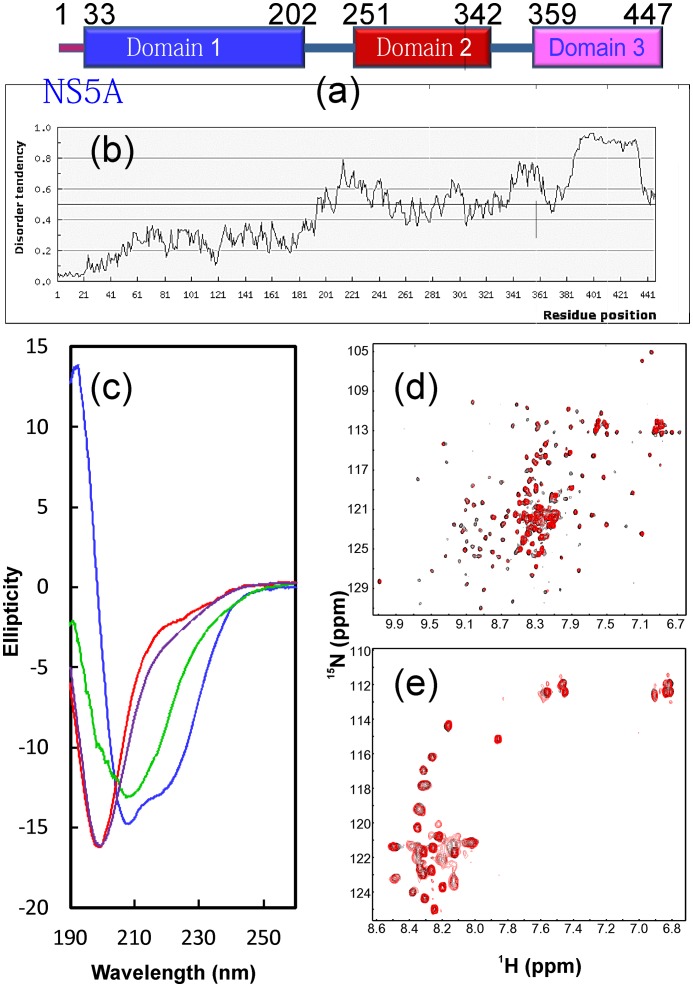
Identification of VAPB domain binding with NS5A. (a). Domain organization of the 447-residue HCV NS5A protein consisting of three domains. (b). Prediction of disorder tendency of the full-length NS5A with IUPred server (http://iupred.enzim.hu/). (c). Far UV CD spectra of NS5A(33–447) (blue); NS5A-D1(33–202) (green); long NS5A-D2(251–380) (red) and long NS5A-D3(300–447) (brown). (d). Superimposition of ^1^H-^15^N NMR HSQC spectra of VAPB(1–195) in the absence of (black) and in the presence of unlabeled NS5A(33–447) (red) at a molar ratio of 1∶2.5 (VAPB/NS5A). (e). Superimposition of ^1^H-^15^N NMR HSQC spectra of VAPB-CC(151–195) in the absence of (black) and in the presence of unlabeled NS5A(33–447) (red) at a molar ratio of 1∶2.5 (VAPB-CC/NS5A).

Based on its domain organization (Figure 2a), we dissected NS5A of a Singapore HCV isolate [55] (Figure S1) into 10 domains/fragments, including NS5A(33–447) only with the first 32 transmembrane residues removed, which were subcloned in either His- or GS-tagged expression vector (Table S1). Except for NS5A-D2-D3 whose expression level was too low to be characterized, all other recombinant proteins were successfully purified for further structural and binding characterizations. As seen in Figure 2c, NS5A(33–447) and NS5A-D1(33–202) have far-UV CD spectra for proteins with well-formed secondary structures, consistent with the fact that the NS5A-D1 domain has a well-formed secondary and tertiary structures [34], [35]. By contrast, the fragments consisting of D2 or/and D3 domains have far-UV CD spectra typical of proteins of being predominantly disordered, consistent with previous reports on NS5A D2 and D3 of other HCV isolates [36]–[39]. We have also assessed the disorder tendency of NS5A (Figure 2b) by IUPred [56]. Very interestingly, while D1 has the lowest and D3 has the highest disorder tendency, D2 appears to be only partially disordered (Figure 2b), thus not a classic intrinsically-unstructured domain. We recently revealed that such partially disordered domains are prone to aggregation or/and undergo large conformational exchanges on the µs-ms time scale [57]. Indeed, probably due to the existence of the dynamic aggregation or/and conformational exchanges in D2 on µs-ms time scale, well-dispersed HSQC spectra could not be obtained for ^15^N-labeled NS5A(33–447) and NS5A-D1(33–202) samples.

### Identification of the VAPB domain binding NS5A

To identify the human VAPB domain for binding with the viral NS5A, we first titrated the ^15^N-labeled VAPB(1–195) sample with the unlabeled NS5A(33–447) protein. As seen in Figure 2c, addition of NS5A(33–447) triggered the disappearance of a subset of HSQC peaks of VAPB(1–195), confirming that NS5A does indeed interact with VAPB. Based on our NMR sequential assignments for both VAPB(1–150) and VAPB(1–125) [46], [51], a close examination reveals that the disappeared and significantly shifted peaks are all from the N-terminal 125 residues which adopt the MSP fold [46]. We also titrated the ^15^N-labeled VAPB-CC sample by NS5A(33–447) but detected no significant change of the HSQC spectra (Figure 2d), indicating that the VAPB coiled coil domain is not physically interacting with NS5A, or the binding is very weak. Taken together, the results suggest that the human VAPB binds to NS5A via its N-terminal MSP domain.

### Identification of NS5A domain binding VAPB

To identify the NS5A domain for binding VAPB, we first titrated ^15^N-labeled VAPB(1–195) and VAPB-CC proteins with the unlabeled NS5A-D1(33–202) proteins with amino acid sequences derived from both Singapore and Con1 isolates (Figure S1), but found no significant change of their HSQC spectra (data not shown), suggesting that D1 is not directly involved in binding with VAPB, or the binding is very weak. Subsequently, we titrated ^15^N-labeled VAPB(1–195) and VAPB-CC proteins with unlabeled NS5A(251–380) and NS5A(313–366) proteins, and again found no significant changes of the HSQC spectra of VAPB(1–195) and VAPB-CC, indicating that D2 as well as the liker between D2 and D3 of NS5A do not physically interact with NS5A, or the binding is too weak to be detected by NMR.

**Figure 3:**
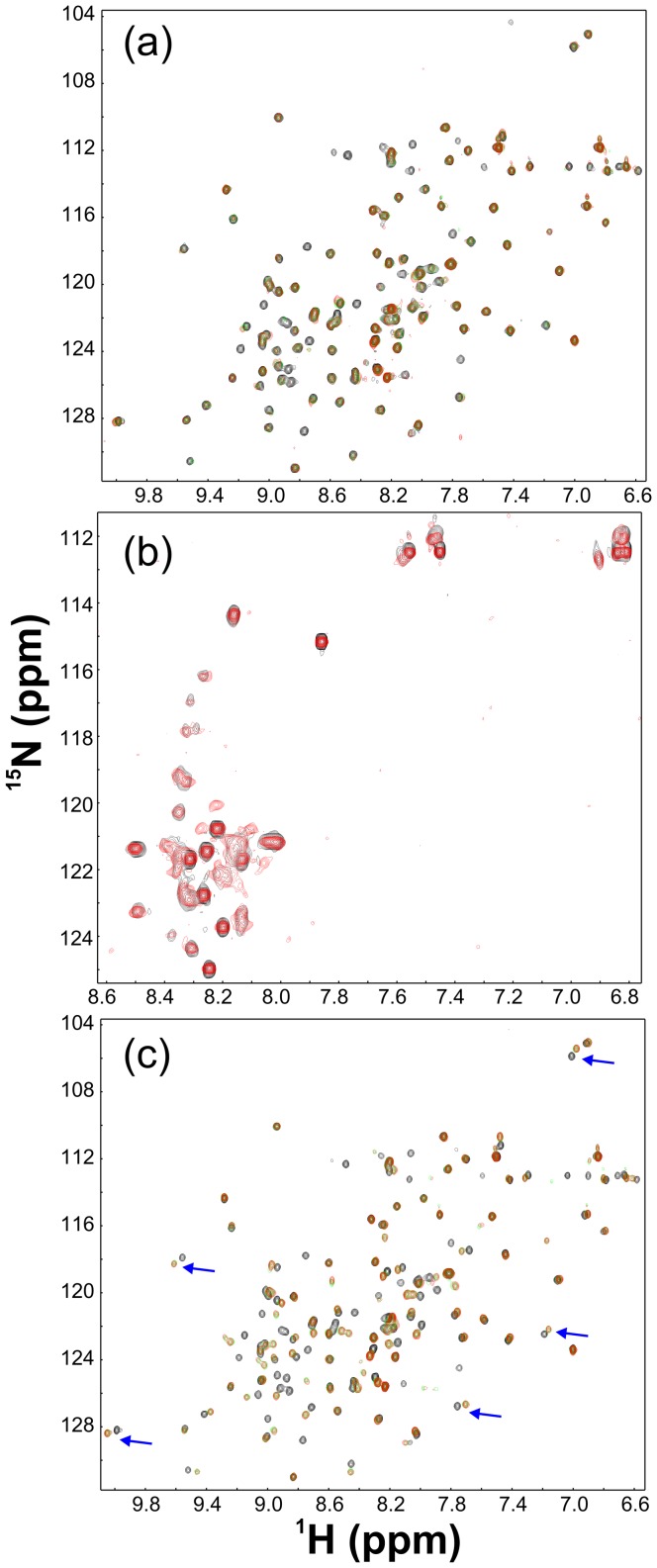
Identification of NS5A domain binding with VAPB. (a). Superimposition of ^1^H-^15^N NMR HSQC spectra of VAPB(1–125) in the absence of (black) and in the presence of unlabeled long NS5A-D3(300–447) at molar ratios of 1∶1.5 (green) and 1∶2.5 (red) (VAPB/NS5A). (b). Superimposition of ^1^H-^15^N NMR HSQC spectra of VAPB-CC(151–195) in the absence of (black) and in the presence of unlabeled long NS5A-D3(300–447) at a molar ratio of 1∶4 (red) (VAPB/NS5A). (c). Superimposition of ^1^H-^15^N NMR HSQC spectra of VAPB(1–125) in the absence of (black) and in the presence of unlabeled NS5A-D3(359–447) at molar ratio of 1∶1 (green) and 1∶2 (red) (VAPB/NS5A).

However, once we titrated ^15^N-labeled VAPB(1–195) with NS5A(300–447) which contains both D3 and the linker between D2 and D3, many HSQC peaks of VAPB(1–195) disappeared or shifted, with a perturbation pattern very similar to that induced by NS5A(33–447) (spectra not shown), implying that NS5A(33–447) and NS5A(300–447) use the similar regions to bind with VAPB. In other words, NS5A-D3 contains at least the majority of the residues critical for binding to VAPB. As such, we titrated the ^15^N-labeled VAPB-MSP(1–125) by the unlabeled NS5A(300–447) and found that the disappeared or shifted HSQC peaks are almost identical to those trigged by NS5A(33–447) (Figure 3a). By contrast, addition of unlabeled NS5A(300–447) caused no significant perturbation on the VAPB-CC HSQC peaks (Figure 3b), again indicating that the VAPB-CC is not physically involved in binding NS5A.

Since NS5A(300–447) protein containing D2 residues has a relatively low solubility and is not suitable for collecting high-quality NMR data for sequential assignment, we further titrated the ^15^N-labeled VAPB(1–125) by NS5A(359–447) containing only VAPB-D3. Remarkably, as shown in Figure 3c, addition of D3 sample not only induced the disappearance of almost the same set of HSQC peaks as induced by NS5A(300–447), but also triggered significant shifts of many extra residues. This implies that the majority of the NS5A residues critical for binding VAPB are located on NS5A-D3. As a consequence, after deleting the D2 and linker residues 300–358 which is not significantly involved in binding, NS5A-D3(359–447) would be expected to have a tighter binding ability to VAPB.

### Structural and binding properties of D3 and its fragments

**Figure 4:**
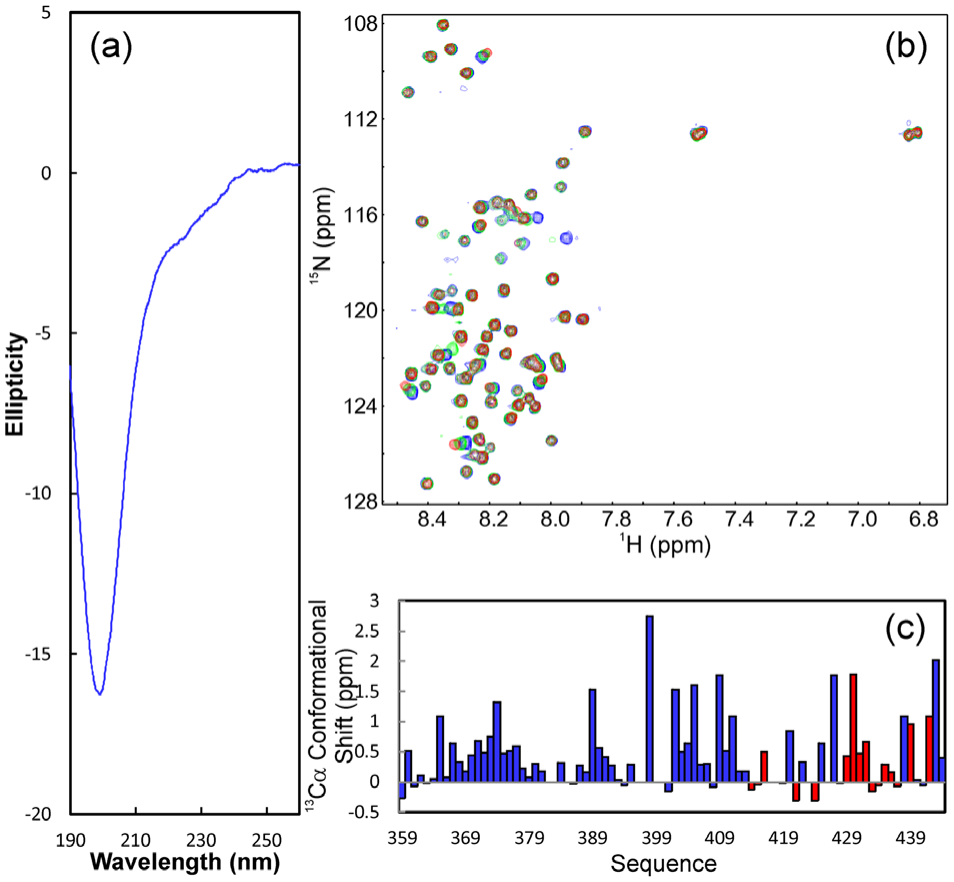
Conformational and binding properties of NS5A-D3. (a). Far UV CD spectrum of NS5A-D3(359–447). (b). Superimposition of ^1^H-^15^N NMR HSQC spectra of NS5A-D3(359–447) in the absence of (blue) and in the presence of unlabeled VAPB-MSP(1–125) at molar ratios of 1∶1 (green) and 1∶2 (red) (D3/MSP). (c). Residue-specific ^13^Cα conformational shift of NS5A-D3(359–447) derived from analysis of triple-resonance heteronuclear NMR spectra including HNCACB and CBCA(CO)NH. Red bars are used to indicate residues undergoing significant shift or disappearance of their HSQC peaks in the presence of unlabeled VAPB-MSP(1–125) at a molar ratio of 1∶2 (D3/MSP).

The recombinant 89-residue NS5A-D3 protein has a far-UV CD spectrum (Figure 4a) typical of highly-unstructured proteins, consistent with previous reports on NS5A-D3 of other HCV isolates [38], [39]. This conclusion is further evident from the very narrow ^1^H-(1.8 ppm) and ^15^N-(19.5 ppm) spectral dispersions of its HSQC spectrum (Figure 4b), which also indicate the absence of a tight tertiary packing in D3. Nevertheless, its relatively high solubility allowed the preparation of a ^15^N-/^13^C-double labeled D3 NMR sample at a protein concentration of 300 µM for collecting triple-resonance NMR data. By analyzing the data, we succeeded in achieving the sequential assignment of NS5A-D3 and obtaining its Cα conformational shifts (Figure 4c). It has been well-established that Cα chemical shift deviations from their random-coil values are very sensitive indicators of protein secondary structures, thus representing a powerful probe for detecting residual secondary structures in unfolded or partially folded proteins [58], [59]. As judged from small but positive Cα conformational shifts over most D3 residues, it can be concluded that the helical conformations are weakly populated over several segments of the sequence (Figure 4c).

**Figure 5:**
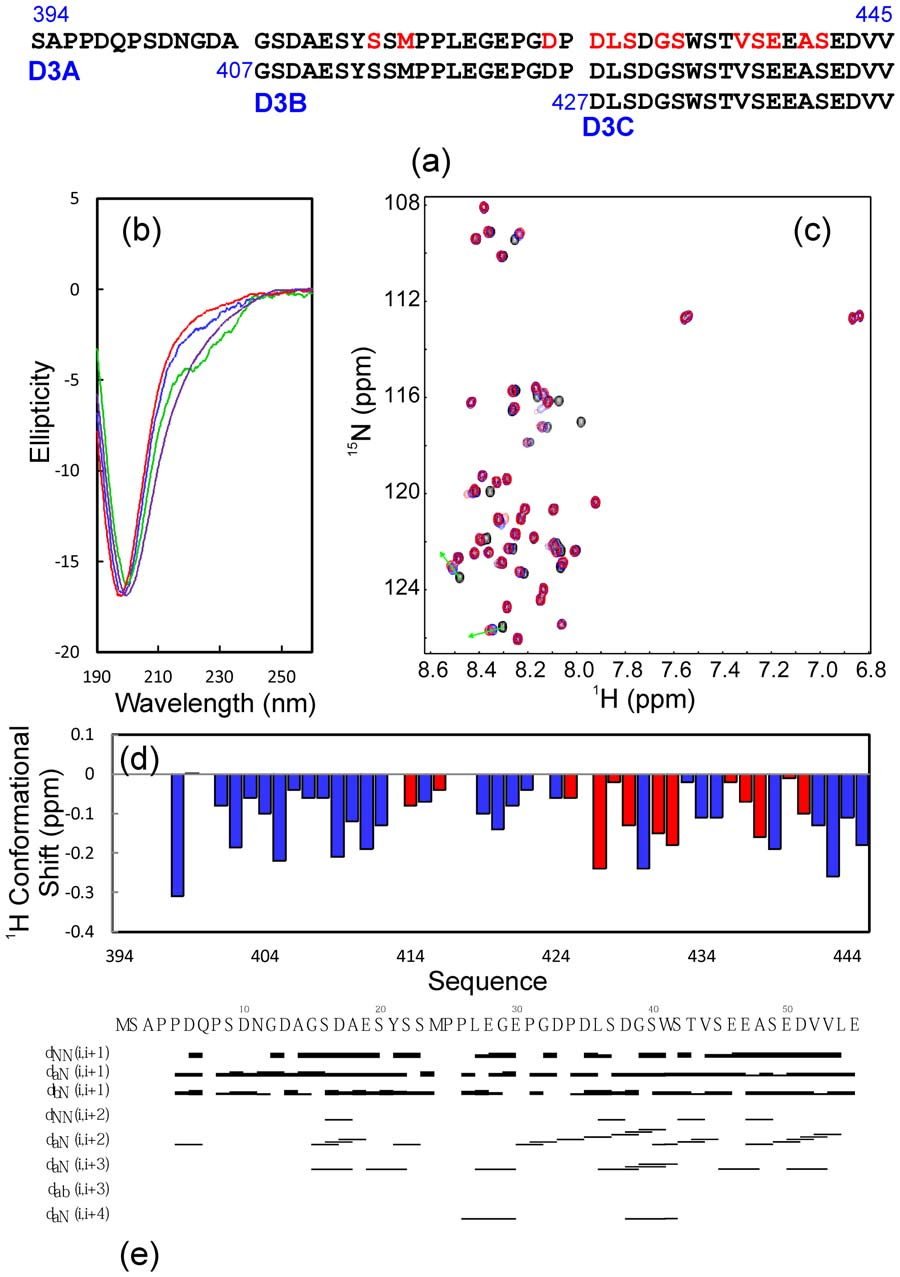
Conformational and binding properties of NS5A-D3 fragments. (a). Amino acid sequence of NS5A-D3A, D3B and D3C. (b). Far-UV CD spectra of NS5A-D3 (blue), D3A (red), D3B (brown) and D3C (green). (c). Superimposition of ^1^H-^15^N NMR HSQC spectra of NS5A-D3A(394–447) in the absence of (black) and in the presence of unlabeled VAPB-MSP(1–125) at molar ratios of 1∶1 (green) and 1∶2 (red) (D3A/MSP). (d). Residue-specific Hα conformational shift of NS5A-D3A(394–447) derived from analysis of three-dimensional ^15^N-edited HSQC-TOCSY spectrum. Red bars are used to indicate residues undergoing significant shift or disappearance of their HSQC peaks in the presence of unlabeled VAPB-MSP(1–125) at a molar ratio of 1∶2 (D3A/MSP). (e). Characteristic NOE connectivities of NS5A-D3A(394–447) defining secondary structures derived from analysis of three-dimensional ^15^N-edited HSQC-NOESY spectrum.

Despite being highly disordered, addition of the unlabeled VAPB(1–125) did lead to significant shifts and disappearance of many D3 HSQC peaks (Figure 4b). Based on the sequential assignment, the perturbed and disappeared residues were identified to be located on the C-terminal half of D3 (Figures 4c and 5a). Therefore, NS5A-D3 is a member of intrinsically-unstructured proteins which is highly-disordered but still functionally-active [60]–[69]. Notably, the binding with VAPB-MSP leads to no significant increase of the spectral dispersion of the D3 HSQC spectra, implying that D3 still remains largely unstructured even upon complexing with VAPB-MSP.

Since the significantly-perturbed residues are located over the C-terminal half of D3 (Figures 4c and 5a), we then subcloned and expressed three further truncated D3 fragments including D3A, D3B and D3C (Figure 5a). As seen in Figure 5b, the 54-residue D3A, 41-residue D3B and 21-residue D3C are all highly disordered in solution as evident from their far-UV CD spectra (Figure 5b). The lacking of a tight tertiary packing of D3A is clearly evident from its narrow HSQC spectral dispersion (Figure 5c). To gain detailed insights into the solution conformation of D3A, we collected a pair of three-dimensional heteronuclear NMR spectra, namely ^15^N-edited HSQC-TOCSY and HSQC-NOESY and subsequently achieved the sequential assignment. As judged from its negative Hα conformational shifts for most residues (Figure 5d), D3A appears to have helical conformations weakly-populated over two segments: Ser401-Ser412 and Asp427-Val445, consistent with Cα conformational shifts of the corresponding residues in D3 (Figure 4c). The existence of two helical segments is further supported by characteristic NOEs (Figure 5e) over the two regions defining the helical conformation, including d_NN(i, i+1)_, d_NN(i, i+2)_, d_αN(i, i+2)_, d_αN(i, i+3)_ and d_αN(i, i+4)_. However, as only two d_αN(i, i+4)_ NOEs are observed, the helical conformations in D3A appear to be mainly dynamic 3_10_-helix, rather than α-helix, consistent with the CD result [56], [57].

D3A is able to bind to the MSP fold as demonstrated by significant shifts of its HSQC peaks upon titrating with the unlabeled VAPB(1–125) (Figure 5c). Based on the sequential assignment, significantly perturbed and disappeared residues has been identified to be mostly located on the second helical segment, while only Ser414 and Met416 are located on the first one (Figure 5d). Strikingly, the binding with the MSP fold again results in no significant increase of the HSQC spectral dispersion, implying that the D3A still remains largely unstructured even upon complexing with VAPB-MSP.

### MSP residues critical for binding NS5A

**Figure 6:**
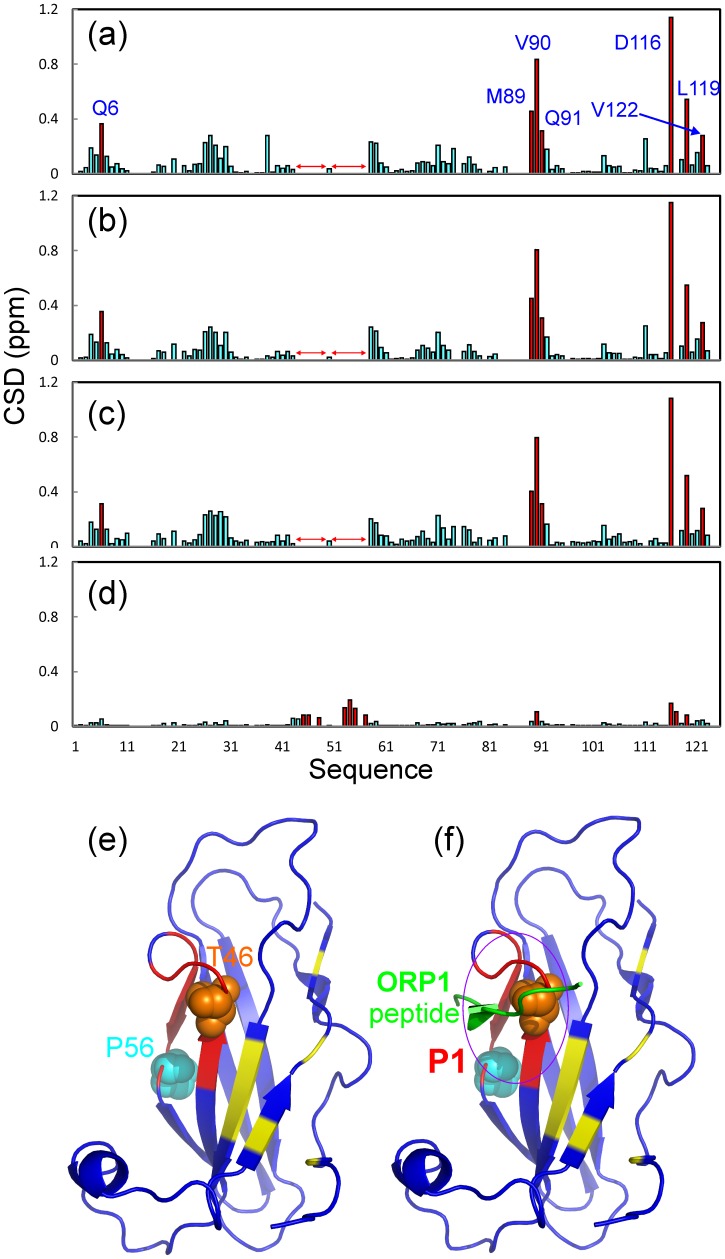
NMR identification of VAPB-MSP residues binding to NS5A-D3 fragments. Residue-specific changes of integrated ^1^H and ^15^N chemical shifts of VAPB-MSP(1–125) in the presence of unlabeled NS5A-D3 (a), D3A (b), D3B (c) and D3C (d) at a molar ratio of 1∶4 (MSP/D3 fragments). Significantly-shifted residues with CSD (chemical shift difference) >1 standard deviations are colored in red and labeled while two regions with disappeared HSQC peaks are indicated by red arrows. (e). Crystal structure of the human VAPB-MSP(1–125) we previously determined (ref. 46) with disappeared (red) and significantly-shifted (yellow) residues mapped out. Two ALS-causing mutants T46I and P56S are displayed in spheres. (f). the FFAT-motif containing ORP1 peptide is further displayed in the structure (ref. 44).

To map out the MSP residues critical for binding NS5A, we titrated the ^15^N-labeled VAPB(1–125) with all four D3 fragments including D3, D3A, D3B and D3C (Figure 5a). Figures 6a–6c present the chemical shift differences (CSD) of the MSP domain upon adding unlabeled D3, D3A and D3B proteins at a molar ratio of 1∶4 (MSP/D3 peptide). Interestingly three peptides trigger almost the same shifting pattern and similar amplitude for the MSP125 residues, suggesting that 41-residue D3B carries almost all residues key for binding the MSP domain. Upon titration, HSQC peaks of the MSP residues Val44-Ala48 and Arg51-Asn57 were found to completely disappear even at a molar ratio of 1∶1 (MSP/D3 peptide). Interestingly, these peaks were also found to disappear in the HSQC spectra of VAPB(1–195) upon titration by NS5A(33–447). On the other hand, upon titration by D3, D3A, D3B, additional HSQC peaks undergo gradual but significant shifts, which include Gln6, Met89-Val90-Gln91, Asp116, Leu119 and Val122.

If the disappeared and significantly shifted residues are mapped back to the VAPB-MSP structure we previously determined [46], they are all located on one side of the β-barrel fold (Figure 6e). Interestingly, the residues with disappeared HSQC peaks upon binding to D3 fragments constitute the interfaces for binding to FFAT-motif containing ORP1 (Figure 6f), and Nir2 peptides [44], [51]. However, additional binding pockets on the MSP fold appear to be involved for binding with NS5A (Figures 6e and 6f). Also it is worthwhile to point out that two ALS-causing mutations, T46I and P56S, are all located over two β-strands with HSQC peaks of most residues disappeared.

On the other hand, unlike D3, D3A and D3B, the 21-residue D3C peptide is no longer able to induce the disappearance of any MSP residues. Instead, as seen in Figure 6d, it is only able to induce peak shifts of some MSP residues of small amplitudes even at a molar ratio of 1∶5 (MSP/D3C). Although the overall perturbation pattern by D3C shares some similarity with those by D3, D3A and D3B, D3C appears to have no significant perturbation on Gln8. These results imply that D3C has a dramatically-reduced binding affinity, as well as smaller contact regions on the MSP fold. This result supports the above conclusion that NS5A-D3 residues Ser414, Met416 and Asp425 are indeed critical for binding VAPB-MSP (Figure 5a).

**Figure 7:**
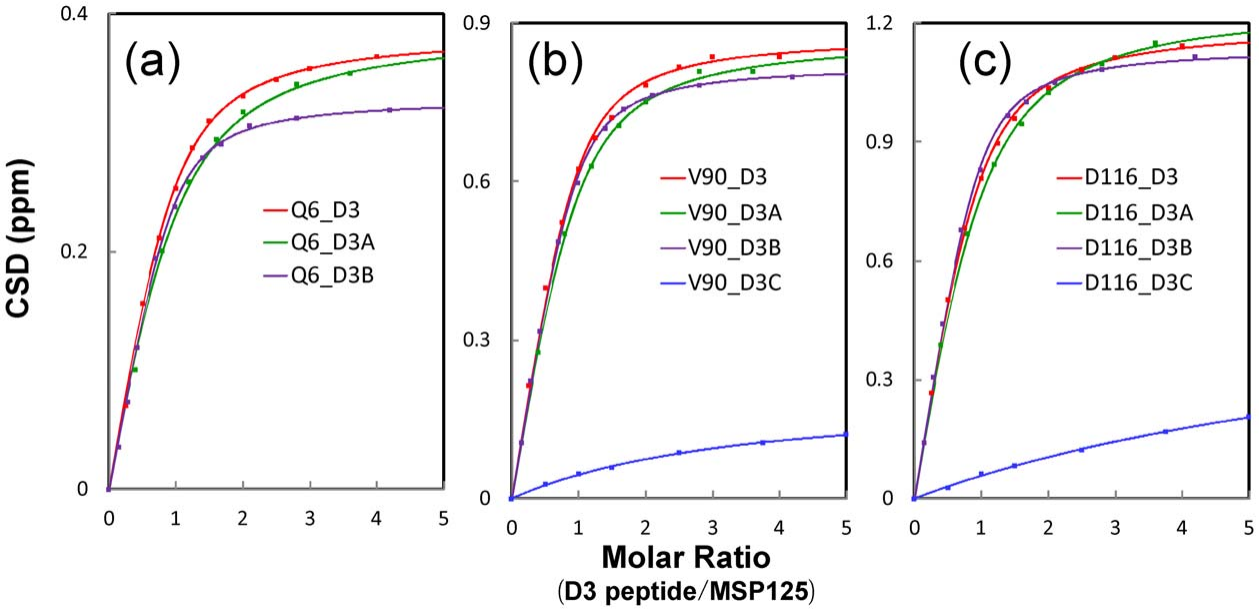
Fitting of chemical shift tracings to obtain dissociation constants (Kd). Experimental (dots) and fitted (lines) values are shown for the integrated ^1^H and ^15^N chemical shift changes of three representative residues: Gln6 (a), Val90 (b) and Asp116 (c) of VAPB-MSP(1–125) induced by gradual addition of NS5A-D3 (red), D3A (green), D3B (purple) and D3C (blue).

Table S2 summarizes NMR titrations in this study. Furthermore, to quantitatively assess the binding, we subsequently fitted the shift tracings of the MSP HSQC peaks to obtain dissociation constants (Kd) as we previously conducted on other systems [70], [71]. The fitting is exemplified in Figure 7 and Kd values are summarized in Table 1. Overall, residue-specific Kd vales titrated by D3, D3A and D3B are very similar, with average Kd values of 4.5, 8.0 and 4.7 µM respectively. For CSDs titrated by D3C, they are very small (Figure 6d) as well as remained largely unsaturated (Figures 7b and 7c) even at a molar ratio of 1∶5 (MSP/D3C). Unfortunately further increase of the molar ratio was not possible due to the low solubility of the D3C peptide. As such, precise Kd values could not be obtained for binding with D3C. This suggests that the residues deleted in D3C also play a key role in binding with MSP.

**Table 1:** Residue-specific Dissociation Constants (Kd) for Binding of Three D3 Fragments to MSP125 as derived from Fitting NMR Titration Results.

	D3		D3A		D3B	
Residues	Kd (µM)	Error	Kd (µM)	Error	Kd (µM)	Error
**Gln6**	6.8	0.6	11.5	1.7	5.5	0.8
**Met89**	5.2	0.6	9.9	0.9	5.3	0.7
**Val90**	4.6	0.7	7.2	0.6	5.0	0.5
**Gln91**	2.4	0.5	7.1	0.9	5.5	0.3
**Asp116**	6.2	0.5	10.2	0.8	5.3	0.6
**Ser117**						
**Leu119**	4.5	0.6	7.7	0.9	4.6	0.6
**Val122**	1.7	0.4	2.4	0.3	1.6	0.6
**Average**	**4.5**		**8.0**		**4.7**	

We also attempted to perform the ITC measurements on the binding of the VAPB(1–125) with different D3C peptides but failed to obtain high-quality data, probably due to the complex binding mode, or/and the fact that many NS5A-D3 residues still remain largely flexible even in the complex.

### Docking model of the D3-MSP complex

We have attempted to co-crystallize VAPB(1–125) with D3 peptides several times but only obtained the crystal of VAPB(1–125) alone, probably due to the relative weak binding affinity, or/and complex binding mode. On the other hand, the extensive disappearance of NMR resonances upon binding with D3 peptides prevented from determining the complex structure by NMR spectroscopy. In this regard, to better capture the binding properties, by using the well-established HADDOCK docking procedure with the NMR titration results, we constructed the models of the MSP-D3B complex, as we previously conducted on other systems [51], [72], [73].

**Figure 8:**
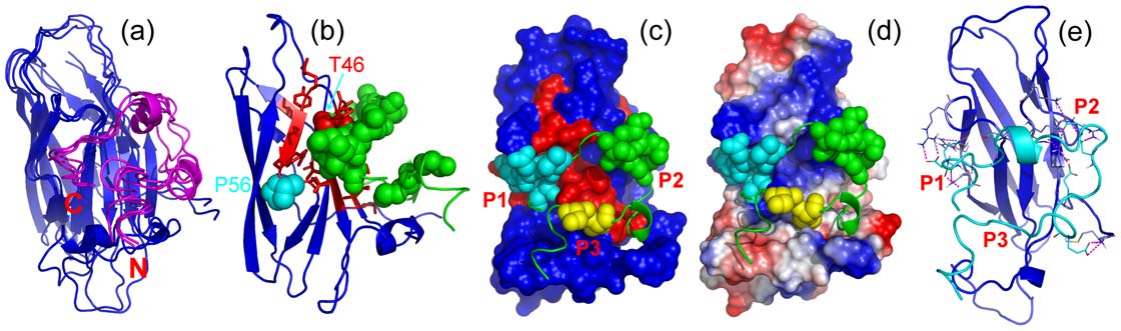
Docking model of MSP-D3C complex. (a). Superimposition of three lowest energy docking models of the MSP-D3C complex. MSP structures are colored in blue and D3C structures in pink. (b). The lowest energy docking model of the MSP-D3C complex, with disappeared and significantly-shifted MSP residues colored in red. Three D3 regions critical for binding with MSP are displayed in spheres. (c). The lowest energy docking model of the MSP-D3C complex, with MSP structure displayed in surface and three discrete D3 regions critical for binding with MSP are displayed in spheres of different colors. Three MSP surface pockets are labeled as P1, P2 and P3 respectively. (d). The lowest energy docking model of the MSP-D3C complex, with the MSP electrostatic potential displayed, with blue, red and grey corresponding to positive, negative and neutral potential values. (e). Hydrogen bonds between D3C and MSP in the complex. Only the residues having the interfacial hydrogen bonds are displayed in sticks and the hydrogen bonds are indicated by the red dashed lines.

Figure 8a shows the superimposition of the three models with the lowest energies in which both MSP and D3B peptides are highly similar. Interestingly, although the initial D3B structure used for docking is an extended conformation without any preferred secondary structures, in all three complex models the D3B peptide acquires helical conformations over two segments: Pro418-Glu420 and Ser429-Trp433. Indeed, these two segments were experimentally characterized to have populated helical conformations, as clearly evident from their conformational shifts (Figure 5d) and NOE connectivities (Figure 5e). As seen in Figure 8b, the significantly-perturbed D3B residues have extensive contacts with the MSP surface where two ALS-causing mutations P56S and T46I are located. In particular, Thr46 appears to have many close contacts with D3B residues Val436 and Ser437. Moreover, three D3B regions (Figure 5a) appear to bind to three distinctive MSP surface patches (Figure 8c): the D3B region over Ser414-Met416 contacts relatively electrostatically neutral pocket of MSP (designated as P3), while other two regions over Asp425-Ser432 and Val436-Ser441 interact with other two electrostatically-positive pockets (designated as P2 and P1 respectively, Figure 8d). As such, there are many hydrogen bonds established between MSP-P1/P2 and D3B residues (Figure 8e). More specifically, over P1 pocket, there are hydrogen bonds between backbone O of NS5A-Glu438 and sidechain NH of MSP-Gln57; between sidechain O of NS5A-Glu438 and sidechain NH of MSP-Arg55; between sidechain O of NS5A-Ser437 and sidechain NH of MSP-Thr46; between sidechain OH of NS5A-Ser437 and backbone O of MSP-Val45; between backbone N of NS5A-Ser437 with sidechain OH of MSP-Thr46; between backbone NH of NS5A-Val436 with sidechain O of MSP-Thr46. For P2 pocket, there are hydrogen bonds between sidechain O of NS5A-Asp425 and sidechain NH of MSP-Arg120; between sidechain O of NS5A-Asp425 and sidechain NH of MSP-Lys87; between sidechain O of NS5A-Asp425 and sidechain NH of MSP-Arg120; between sidechain O of NS5A-Asp427 and sidechain NH of MSP-Lys118; between sidechain O of NS5A-Asp427 and sidechain NH of MSP-Lys85.

### NS3A competes with EphA4 in binding VAPB

**Figure 9:**
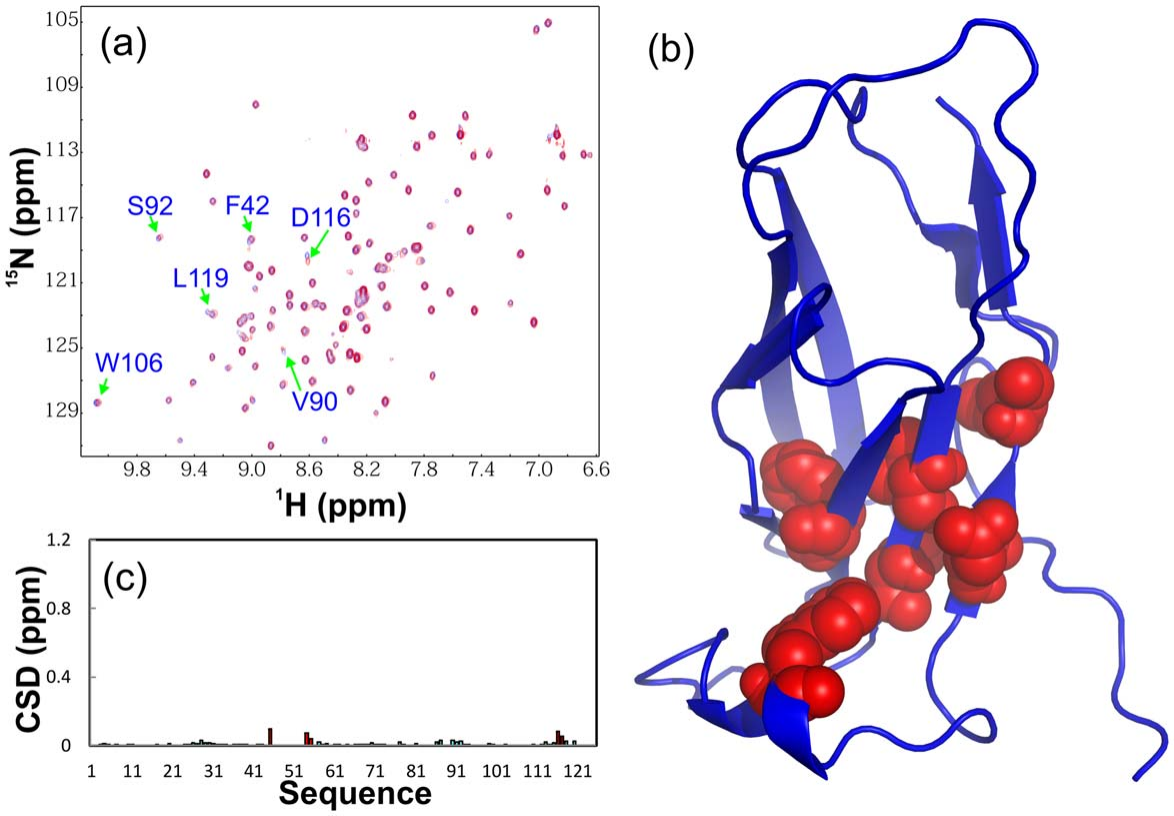
NS5A competes with EphA4 in binding with MSP. (a). Superimposition of ^1^H-^15^N NMR HSQC spectra of VAPB-MSP(1–125) saturated with the pre-existence of D3C at a molar ratio of 1∶2 (MSP/D3C), in the absence of (blue) and in the presence of 181-residue EphA4 ligand binding domain at a molar ratio of 1∶10 (red) (MSP/EphA4). Significantly-shifted residues are labeled. (b). Crystal structure of the human VAPB-MSP(1–125) with significantly-shifted residues displayed in red spheres. (c). Residue-specific changes of integrated ^1^H and ^15^N chemical shifts of VAPB-MSP(1–125) T46I mutant in the presence of unlabeled NS5A-D3C at a molar ratio of 1∶6 (MSP/D3C). Significantly-shifted residues with CSD (chemical shift difference) >1 standard deviation are colored in red.

NMR titration and docking results all reveal that the D3B peptide is able to occupy the MSP surface regions which are also critical for binding to EphA4 receptor as we previously characterized [51]. To confirm this, we first saturated the VAPB-MSP(1–125) by D3B at a molar ratio of 1∶4 (MSP/D3B). Subsequently the EphA4 ligand-binding domain (LBD) was gradually titrated into this sample. Unlike the previous observation that addition of EphA4 LBD to VAPB-MSP even at a molar ratio of 1∶2 started to trigger peak shift and disappearance of the MSP residues [51], in the pre-existence of D3B peptide, only several MSP peaks were observed to shift slightly when the molar ratio reached 1∶10 (MSP∶EphA4) (Figure 9a). Interestingly, the shifted residues are all located on the MSP surface perturbed by both D3B and EphA4 (Figure 9b). These results thus suggest that in the pre-existence of D3B, EphA4 at low concentration is not able to bind to VAPB-MSP but at high concentrations, EphA4 will start to displace the D3B peptide from binding with the MSP fold. Therefore, NS5A does compete with EphA4 in binding with VAPB-MSP.

We also assessed the binding of D3B to the ALS-causing T46I mutant, which we previously demonstrated to have a reduced affinity to EphA4. Remarkably, as shown in Figure 9b, even at a molar ratio up to 1∶6 (T46I/D3B), only a small set of T46I HSQC peaks were found to shift slightly of very small amplitude. As the shift tracings remain highly unsaturated, it is impossible to fit out the CSD tracings to obtain Kd values. Nevertheless, the results clearly indicate that T46 does play an important role in binding to NS5A-D3 as also reflected by the docking model.

## Discussion

Formation of the host membrane associated replication complexes appears to be a common property for RNA viruses such as HCV [74], [75]. Although currently little is known about the structure of the complexes, it is anticipated that studies of RNA virus replication machineries will have a critical impact on antiviral drug development due to their specific roles in virus replication [74], [75]. On the other hand, despite being reported 8 years ago, there has been no *in vitro* structural and binding characterization on the NS5A-VAP interactions to date. Therefore, to bridge the gap, we initiated a systematic investigation on the NS5A-VAPB interactions by first dissecting both VAPB and NS5A proteins into 14 domains/fragments, followed by extensive structural and binding characterizations with heteronuclear NMR spectroscopy which is powerful in detecting even very weak binding. Our study reveals that the C-terminal half of NS5A-D3 is indeed capable of binding to VAPB-MSP, with an average dissociation constant (Kd) of ∼5 µM.

CD and NMR characterizations demonstrate that NS5A-D3 of the Singapore HCV isolate is also highly disordered without any tight tertiary packing and stable secondary structure. Nevertheless, it is active in binding to the VAPB-MSP domain, thus demonstrating that it is an intrinsically unstructured domain. On the other hand, by analyzing triple-resonance heteronuclear NMR spectra, NMR assignments have been achieved, which allows identification of helical conformations weakly populated over NS5A-D3. Importantly, the NS5A-D3 residues critical for binding to VAPB-MSP have been mapped out to be clustered over three relatively discrete regions, which is further confirmed by three truncated fragments. Remarkably, finally we obtain a 41-residue fragment D3B, which has N-terminal 48 residues deleted but retains almost the same binding affinity and mode as the 89-residue D3.

Very surprisingly, even upon binding to the VAPB-MSP domain, D3 peptides are still lacking of very tight tertiary packing required to manifest large spectral dispersions of the HSQC spectrum. In fact, several years ago, we have found a similar phenomenon on a transcriptional activator ApLLP for long-term memory formation, which is not only highly disordered in the free state, but intriguingly remains largely unstructured even upon forming a complex with DNA [67]. Similarly, no high-quality ITC profile could be obtained on the ApLLP-DNA interaction. Now it is starting to be recognized that this phenomenon in fact exists in a large number of protein-protein, protein-DNA complexes involved in intrinsically unstructured proteins, thus being designated as “Fuzzy Complex” [68], [69]. The fuzzy or dynamic property for the NS5A-VAPB complex is most likely to result from the fact that even in D3B, many residues are not significantly engaged in binding to VAPB-MSP and thus remain flexible (Figure 5a). Here it is tempted to speculate that the dynamic feature for the NS5A-VAPB interaction may in fact facilitate the functional actions of the HCV RNA replication machinery which is expected to undergo dynamic assembly and disassembly.

Previously we have determined the crystal structure of the human VAPB-MSP domain but in the present study, we failed to obtain the co-crystal of the MSP-D3B complex, probably due to the relatively-weak binding affinity or/and dynamic nature of this fuzzy complex. The crystallization may be significantly interfered by the presence of large regions that remain disordered in the fuzzy complexes. Nevertheless, by use of NMR spectroscopy, we have successfully mapped out both NS5A and VAPB residues key for forming the NS5A-VAPB complex, which thus allow the construct of a complex model by use of a well-established NMR constraint-based docking procedure. In the complex, the NS5A-D3 binds to one side of the MSP fold which was also characterized to interact with FFAT-containing peptides and Eph receptor [44], [46], [51]. More precisely, three D3 regions appear to bind three MSP surface pockets respectively. Pockets 1 and 2 are electrostatically positive while pocket 3 is relatively electrostatically neutral. Interestingly, ALS-causing mutation P56S is located in the pocket 1 while T46I sites in between pocket 1 and pocket 2.

We experimentally demonstrated that the NS5A-D3 indeed has overlapped binding regions with EphA4 on VAPB-MSP, and ALS-causing mutation T46I dramatically abolishes the binding affinity of the MSP domain to NS3A-D3. This observation may rationalize a recent report that a chronic HCV patient surprisingly developed an ALS-like syndrome [76], [77]. We speculate here that in some chronic HCV patients, the NS5A protein may abnormally accumulate which consequently perturbs the physiological functions of the VAP proteins, in a similar way as the T46I mutation, which does not severely disrupt the MSP structure but considerably reduces the binding affinity to EphA4 [51]. As such, our present results strengthen our previous speculation that the VAPB-MSP domain might be a key convergent point for signaling pathways important for ALS pathogenesis [51]. In the future, it would be of significant interest to explore the design of peptides/small molecules to target the intrinsically unstructured NS5A-D3 or/and its well-folded binding partner, VAPB-MSP as previously demonstrated on other systems [78], [79].

## Materials and Methods

### Cloning, expression and purification of VAPB/NS5A domains/fragments

As summarized in Table S1, in the present study we have cloned four human VAPB and 10 HCV NS5A dpmains/fragments. Briefly the DNA fragment encoding the human VAPB(1–195) was amplified from HeLa cell cDNA library by using two designed primers, which was subsequently cloned into a modified pET32a vector (Novagen). Subsequently the VAPB-MSP(1–125), VAPB(1–150) and VAPB-CC(151–195) were amplified by using designed primers and sub-cloned in modified pET32a vector. Similarly DNA fragments encoding different NS5A domains/fragments were amplified from cDNA for HCV genotype 1b strain S1 and strain Con1, which were sub-cloned in expression vectors (Table S1).

All the expression vectors were transformed into *Escherichia coli* BL21 (DE3) Star (invitrogen) cells. For expression of recombinant proteins, cells were grown in Luria-Bertani (LB) medium in the presence of ampicillin (100 µg/ml) at 37°C to reach the absorbance of 0.6 at 600 nm and subsequently induced with respective optimized IPTG concentrations. Harvested cells were resuspended and lysed by sonication in lysis buffer (50 mM Tris, 500 mM NaCl, 10% glycerol, 20 mM imidazole, 10 mM 2-mercaptoethanol, pH 7.5) containing protease inhibitor cocktail (Roche). His-tagged proteins were purified by Ni^2+^-affinity chromatography (Qiagen) while GST-fused proteins were purified by affinity chromatography with glutathione-Sepharose 4B beads (Pharmacia Biotech) under native conditions. The VAPB and NS5A proteins were released from the fused tags by in-gel thrombin cleavage, which were further purified either by FPLC on Superdex 75/Superdex 200 columns (Pharmacia Biotech), or by HPLC on a RP (reverse phase) C18 column (Vydac) (Table S1).

The production of the isotope-labeled VAPB and NS5A domain/fragment proteins for NMR studies followed a similar procedure except that the bacteria were grown in M9 medium with the addition of (^15^NH_4_)_2_SO_4_ for ^15^N labeling and (^15^NH_4_)_2_SO_4_/(^13^C)-glucose for ^15^N-/^13^C double labeling (67–70). The purity of all protein samples was checked by the SDS-PAGE gel and their molecular weights were verified by a Voyager STR matrix-assisted laser desorption ionization time-of-flight-mass spectrometer (Applied Biosystems). The identities of NS5A-D3/D3A and VAPB(1–125)/(1–150) were further confirmed by NMR assignments. The concentration of protein samples was determined by the spectroscopic method in the presence of denaturant [70]–[73], [80].

### Circular dichroism (CD) and NMR experiments

All CD experiments were carried out in a Jasco J-810 spectropolarimeter (Jasco Corporation, Tokyo, Japan) as previously described at 25°C [70]–[73]. The protein concentration is 20 µM in 2 mM phosphate buffer (pH 6.8) for all far-UV CD experiments.

NMR samples were prepared in 10 mM phosphate buffer in the presence of 10 mM DTT (pH 6.8). All NMR data were collected at 25°C on an 800-MHz Bruker Avance spectrometer equipped with a shielded cryoprobe as described before [70]–[73]. For HSQC characterization, samples were prepared at a protein concentration of 100 µM. For sequential assignments of NS5A-D3, triple-resonance NMR experiments including HNCACB and CBCA(CO)NH were acquired on a double-labeled sample at a protein concentration of ∼300 µM while for sequential assignments of NS5A-D3A, three-dimensional heteronuclear NMR experiments including HSQC-TOCSY and HSQC-NOESY were acquired on a ^15^N-labeled sample at a protein concentration of ∼500 µM. NMR data were processed with NMRpipe [81] and analyzed with NMRview [82].

### ITC and NMR characterization of binding interactions

ITC experiments were performed using a Microcal VP ITC machine as we previously conducted [72], [73]. Titrations of NS5A peptides to the VAPB-MSP domain were conducted in 10 mM phosphate buffer at pH 7.5. Usually the VAPB-MSP samples were placed in the cell while the NS5A peptides were taken in syringe. The samples were degassed for 15 min to remove bubbles before titrations were initiated.

For NMR HSQC characterization of the binding interactions between NS5A and VAPB fragments/domains, two-dimensional ^1^H-^15^N HSQC spectra of the ^15^N-labeled proteins were acquired at a protein concentration of 50 µM in the absence or presence of the unlabeled binding partners at different molar ratios. By superimposing the HSQC spectra at different molar ratios, the shifted or disappeared HSQC peaks could be identified and further assigned to the corresponding residues as we previously described [51], [67]–[70]. The degree of perturbation was reflected by an integrated chemical shift difference (CSD) calculated by the formula ((Δ^1^H)^2^+(Δ^15^N)^2^/5)^1/2^ [73]. The CSD tracings were fitted by using the one binding site model [72] to obtain residue-specific dissociation constants (Kd); which are summarized in Table 1.

### Molecular docking of the D3B-MSP complex

The models of the complex structure between NS5A-D3B and VAPB-MSP were constructed as we previously described on other systems [51], [72], [73], by use of the HADDOCK software [83] in combination with CNS [84], which makes use of chemical shift perturbation data to derive the docking while allowing various degrees of flexibility. The docking procedure was performed by three steps as follows: first, randomization and rigid body energy minimization; second, semi-flexible simulated annealing; and third, flexible explicit solvent refinement. Briefly, all D3B (Figure 5a) and MSP (Figure 6c) residues with HSQC peaks disappeared or significantly shifted were set to be “active” residues, whereas neighbors of active residues were defined as “passive” residues according to HADDOCK definition. The crystal structure of VAPB-MSP (3IKK) we previously determined [46] was used here while an extended D3B structure was utilized for docking. One thousand structures were generated during the rigid body docking, and the best 50 structures were selected for semi-flexible simulated annealing, followed by water refinement. Three structures with the lowest energies were selected for detailed analysis and display by the PyMOL molecular graphics system (W. L. DeLano, DeLano Scientific LLC, San Carlos, CA).

## Supporting Information

Figure S1 **Sequence Alignment.**
(TIF)

Table S1 **Differentially-dissected Domains/Fragments of VAPB and NS5A.**
(DOCX)

Table S2 **Summary of NMR Titrations.**
(DOCX)
