# Supplementary figures and images for: Intrinsically Unstructured Domain 3 of Hepatitis C Virus NS5A Forms a “Fuzzy Complex” with VAPB-MSP Domain Which Carries ALS-Causing Mutations
Source: PLoS One. 2012 Jun 13;7(6):e39261. doi: 10.1371/journal.pone.0039261 (PMC3374797; doi:10.1371/journal.pone.0039261)

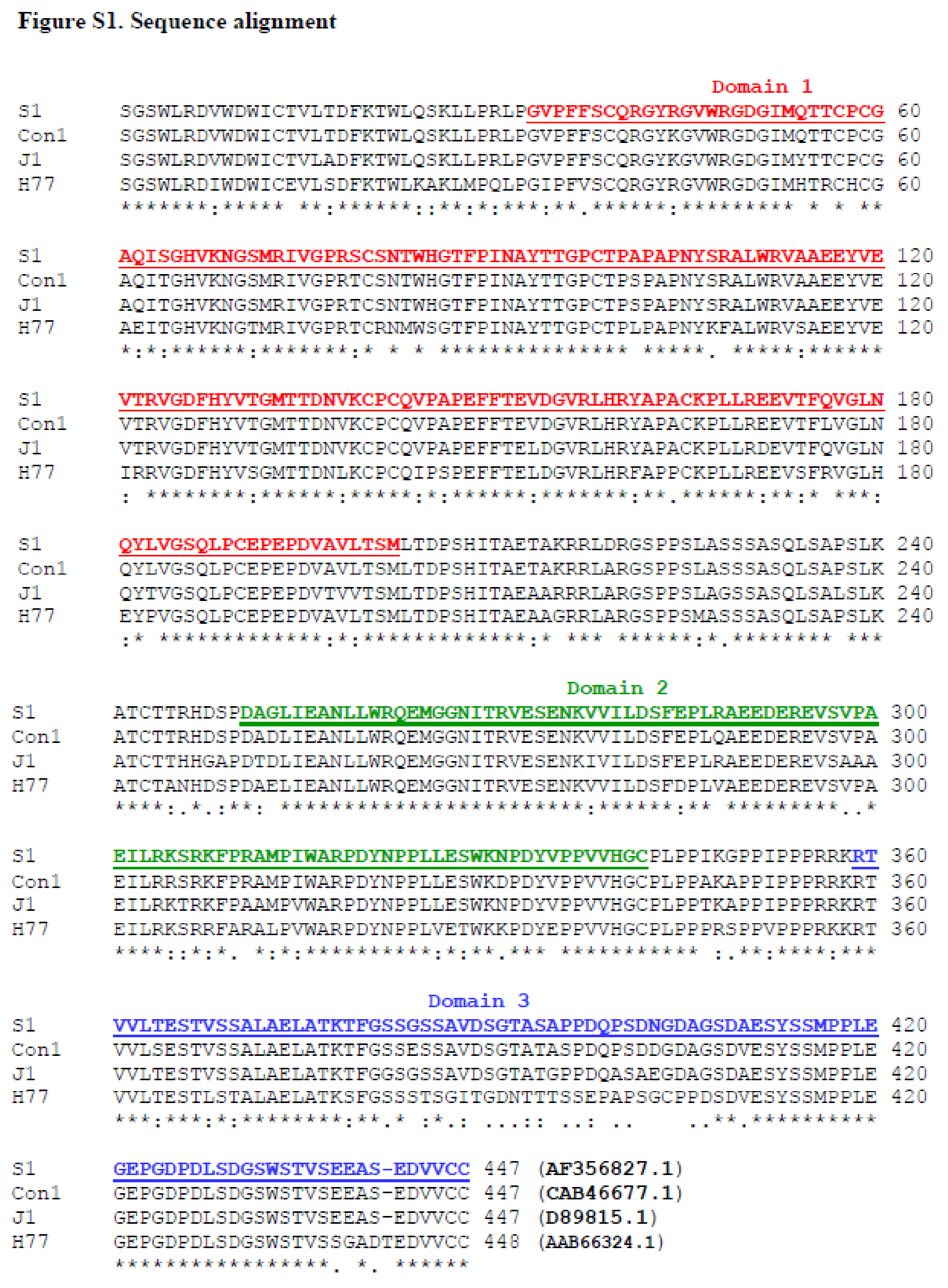

Supplement: Figure S1 — Sequence Alignment. (TIF) [file pone.0039261.s001.tif]
