# Supplementary material for: Intrinsically Unstructured Domain 3 of Hepatitis C Virus NS5A Forms a “Fuzzy Complex” with VAPB-MSP Domain Which Carries ALS-Causing Mutations
Source: PLoS One. 2012 Jun 13;7(6):e39261. doi: 10.1371/journal.pone.0039261 (PMC3374797; doi:10.1371/journal.pone.0039261)
Supplement: Table S1 — Differentially-dissected Domains/Fragments of VAPB and NS5A. (DOCX) [file pone.0039261.s002.docx]

**Table S1. Differentially-dissected Domains/Fragments of VAPB and NS5A**

|  | **Construct** | **Cloning Vector** | **Residues** | **Purification** |
| --- | --- | --- | --- | --- |
| **VAPB** | VAPB | N-His-tagged (pET32a) | 1-195 | Ni^2+^-affinity followed by FPLC |
|  | Long VAPB-MSP | N-His-tagged (pET32a) | 1-150 | Ni^2+^-affinity followed by FPLC |
|  | VAPB-MSP | N-His-tagged (pET32a) | 1-125 | Ni^2+^-affinity followed by FPLC |
|  | VAPB-CC | N-His-tagged (pET32a) | 151-195 | Ni^2+^-affinity followed by HPLC |
|  |  |  |  |  |
| **NS5A** | NS5A | N-His-tagged (pET32a) | 33-445 | Ni^2+^-affinity followed by FPLC |
|  | NS5A-D1 | N-His-tagged (pET32a) | 33-202 | Ni^2+^-affinity followed by FPLC |
|  | NS5A-D2-D3 | N-His-tagged (pET32a) | 251-445 | very low expression level |
|  | Long NS5A-D2 | N-His-tagged (pET32a) | 251-380 | Ni^2+^-affinity followed by FPLC |
|  | NS5A-D2-D3 Loop | N-GST-tagged (pGEX-4T1) | 313-366 | Glutathione-affinity followed by HPLC |
|  | Long NS5A-D3 | C-His-tagged (pET28a) | 300-445 | Ni^2+^-affinity followed by HPLC |
|  | NS5A-D3 | C-His-tagged (pET22b) | 359-445 | Ni^2+^-affinity followed by HPLC |
|  | NS5A-D3A | C-His-tagged (pET22b) | 394-445 | Ni^2+^-affinity followed by HPLC |
|  | NS5A-D3B | N-GST-tagged (pGEX-4T1) | 407-445 | Glutathione-affinity followed by HPLC |
|  | NS5A-D3C | N-GST-tagged (pGEX-4T1) | 427-445 | Glutathione-affinity followed by HPLC |
