# Supplementary material for: Intrinsically Unstructured Domain 3 of Hepatitis C Virus NS5A Forms a “Fuzzy Complex” with VAPB-MSP Domain Which Carries ALS-Causing Mutations
Source: PLoS One. 2012 Jun 13;7(6):e39261. doi: 10.1371/journal.pone.0039261 (PMC3374797; doi:10.1371/journal.pone.0039261)
Supplement: Table S2 — Summary of NMR Titrations. (DOCX) [file pone.0039261.s003.docx]

**Table S2. Summary of NMR Titrations**

| **NS5A Fragment** | **VAPB** **Fragment** | **Interaction** (Yes/No) | **Figure** |
| --- | --- | --- | --- |
| NS5A(33-447) | ^15^N-VAPB(1-195) | Yes | Fig. 2c |
| NS5A(33-447) | ^15^N-VAPB-CC(151-195) | No | Fig. 2d |
| NS5A-D1(33-202) | ^15^N-VAPB(1-195) | No | Not shown |
| NS5A-D1(33-202) | ^15^N-VAPB-CC(151-195) | No | Not shown |
| NS5A(251-380) | ^15^N-VAPB(1-195) | No | Not shown |
| NS5A(251-380) | ^15^N-VAPB-CC(151-195) | No | Not shown |
| NS5A(313-366) | ^15^N-VAPB(1-195) | No | Not shown |
| NS5A(313-366) | ^15^N-VAPB-CC(151-195) | No | Not shown |
| Long NS5A-D3(300-445) | ^15^N-VAPB(1-195) | Yes | Not shown |
| Long NS5A-D3(300-445) | ^15^N-VAPB-MSP(1-125) | Yes | Fig. 3a |
| Long NS5A-D3(300-445) | ^15^N-VAPB-CC(151-195) | No | Fig. 3b |
| NS5A-D3(359-445) | ^15^N-VAPB-MSP(1-125) | Yes | Fig. 3c |
| ^15^N-,^13^C-NS5A-D3 | VAPB-MSP(1-125) | Yes | Fig. 4b |
| ^15^N-NS5A-D3A(394-445) | VAPB-MSP(1-125) | Yes | Fig. 5c |
| NS5A-D3(359-445) | ^15^N-VAPB-MSP(1-125) | Yes | Fig. 6a. To map VAP residues |
| NS5A-D3A(394-445) | ^15^N-VAPB-MSP(1-125) | Yes | Fig. 6b. To map VAP residues |
| NS5A-D3B(407-445) | ^15^N-VAPB-MSP(1-125) | Yes | Fig. 6c. To map VAP residues |
| NS5A-D3C(427-445) | ^15^N-VAPB-MSP(1-125) | Yes/Weak | Fig. 6d. |
| NS5A-D3B(407-445) | ^15^N-VAPB-MSP(1-125) T46I | Yes/Weak | Fig. 9c. |
